# Growth–defense trade‐offs promote habitat isolation between recently‐diverged species

**DOI:** 10.1002/ece3.11609

**Published:** 2024-06-30

**Authors:** Julia G. Harenčár, Diego Salazar‐Amoretti, Carlos García‐Robledo, Kathleen M. Kay

**Affiliations:** ^1^ Ecology and Evolutionary Biology Department University of California Santa Cruz California USA; ^2^ Department of Biological Sciences Binghamton University Binghamton New York USA; ^3^ Department of Ecology and Evolutionary Biology University of Connecticut Storrs Connecticut USA

**Keywords:** *Costus*, habitat adaptation, herbivore escape, herbivory, reproductive isolation, species maintenance, tropical plants

## Abstract

Trade‐offs are crucial for species divergence and reproductive isolation. Trade‐offs between investment in growth versus defense against herbivores are implicated in tropical forest diversity. Empirically exploring the role of growth–defense trade‐offs in closely related species' reproductive isolation can clarify the eco‐evolutionary dynamics through which growth–defense trade‐offs contribute to diversity. *Costus villosissimus* and *C. allenii* are recently diverged, interfertile, and partially sympatric neotropical understory plant species primarily isolated by divergent habitat adaptation. This divergent adaptation involves differences in growth rate, which may constrain investment in defense. Here, we investigate growth–defense trade‐offs and how they relate to the divergent habitat adaptation that isolates these species. We characterize leaf toughness and chemistry, evaluate the feeding preferences of primary beetle herbivores in controlled trials and field‐based experiments, and investigate natural herbivory patterns. We find clear trade‐offs between growth and defense: slower‐growing *C. allenii* has tougher leaves and higher defensive chemical concentrations than faster‐growing *C. villosissimus*. *Costus villosissimus* has rapid growth‐based drought avoidance, enabling growth in drier habitats with few specialist herbivores. Therefore, growth–defense trade‐offs mediate synergistic biotic and abiotic selection, causing the divergent habitat adaptation that prevents most interspecific mating between *C. villosissimus* and *C. allenii*. Our findings advance understanding of ecological speciation by highlighting the interplay of biotic and abiotic selection that dictates the outcome of trade‐offs.

## Graphical abstract

Growth–defense trade‐offs have long been implicated in tropical plant diversity, but how do these tradeoffs contribute to reproductive isolation? This study characterizes growth–defense trade‐offs between two recently diverged tropical plants and explore their role in maintaining strong habitat isolation. We describe how abiotic and biotic selection act synergistically via growth–defense trade‐offs to promote habitat isolation.

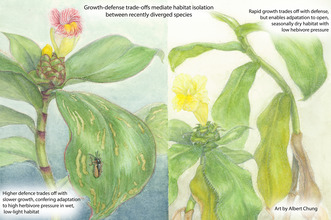

## Resúmen

Los compromisos son cruciales para la divergencia de especies y el aislamiento reproductivo. El compromiso entre crecimiento y defensa contra los herbívoros juega un papel central en la diversidad de los bosques tropicales. Explorar empíricamente el papel de las compromisos entre crecimiento y defensa en el aislamiento reproductivo de especies recientemente divergentes puede aclarar la dinámica ecoevolutiva a través de la cual los compromisos entre crecimiento y defensa contribuyen a la diversidad. *Costus villosissimus* y *C. allenii* son dos especies de plantas de sotobosque neotropical que divergieron recientemente, son interfértiles y parcialmente simpátricas, aisladas principalmente por adaptación a hábitates divergentes. Esta adaptación divergente implica diferencias en la tasa de crecimiento, lo que puede limitar la inversión en defensas. Aquí investigamos las compensaciones entre crecimiento y defensa y cómo se relacionan con la adaptación divergente del hábitat que aísla a estas especies. Caracterizamos la dureza y la química de las hojas, evaluamos las preferencias alimenticia de los escarabajos herbívoros en ensayos controlados y experimentos de campo, e investigamos los patrones de herbivoría natural. Encontramos compromisos entre crecimiento y defensa: *C. allenii*, es de crecimiento lento, tiene hojas más duras y concentraciones de defensas químicas más altas que en *C. villosissimus*, que presenta un crecimiento rápido. *Costus villosissimus* evita la sequía usando una estrategia basada en crecimiento rápido que permite el crecimiento en hábitats más secos y con pocos herbívoros especializados. Por lo tanto, las compromisos entre crecimiento y defensa median presiones de selección bióticas y abióticas sinérgicas que causan la adaptación divergente del hábitat que previene la mayoría de entrecruzamientos interespecíficos entre *C. villosissimus* y *C. allenii*. Nuestros hallazgos avanzan en la comprensión de la especiación ecológica al resaltar la interacción de la selección biótica y abiótica que dicta el resultado de las compromisos.

## 1 INTRODUCTION

Trade‐offs in resource allocation and life history shape and constrain the phenotypes of organisms and can influence species formation and maintenance (Antonovics, 1976). Plants face trade‐offs between allocating resources to growth and reproduction versus structural and chemical defenses against enemies (Coley et al., 1985; Endara & Coley, 2011; Kursar & Coley, 2003). Plants that grow quickly are often poorly defended, whereas slow‐growing plants generally make tough, well‐defended leaves (Coley et al., 1985; Herms & Mattson, 1992; Simms & Rausher, 1987). Pest pressure, resource availability, and phylogenetic constraints determine a plant species' position along a spectrum of investment in growth versus defense (Coley et al., 1985; Wink, 2003). These growth–defense trade‐offs have been invoked to help explain diverse ecological and evolutionary phenomena, such as species turnover across environmental clines, large and small‐scale patterns of habitat specialization, and ecological speciation (Becerra, 2007; Coley et al., 1985; Endara & Coley, 2011; Vleminckx et al., 2018).

Growth–defense trade‐offs are thought to promote the remarkable diversification of tropical plants, but potential mechanisms for a role in reproductive isolation remain unclear. Most studies on growth–defense trade‐offs and tropical plant diversity do not address how species diverged; instead, they focus on fully diverged species that are likely no longer interfertile. For example, co‐occurring tropical plant species, regardless of relatedness, have greater divergence in defense investment than expected for randomly assembled communities, implicating differences in growth–defense strategies in species reproductive isolation (Becerra, 2007; Coley et al., 2018; Daly et al., 2022; Kursar et al., 2009; Salazar et al., 2016; Sedio et al., 2017; Vleminckx et al., 2018). Several phylogenetic studies of chemical defenses in tropical genera also find no phylogenetic signal, reflecting that closely related species are often more different in chemical defense composition than predicted by phylogenetic relatedness alone (Becerra, 1997; Coley et al., 2018; Endara et al., 2017; Kursar et al., 2009; Salazar et al., 2016). However, these phylogenetic studies do not test potential mechanisms through which differences in growth and defense investment can facilitate reproductive isolation and speciation.

Growth–defense trade‐offs may promote reproductive isolation by preventing one species from simultaneously optimizing both a growth‐ and a defense‐oriented strategy in an environment containing relatively discrete niches. Discrete niches can cause divergent adaptation that reduces mating frequency or confers lower hybrid fitness, contributing to pre‐ and postzygotic reproductive isolation (Marquis et al., 2016; Sobel et al., 2010). Plant taxa may initially diverge across a geographic gradient to conditions that select more strongly for either growth or defense, as has been observed in studies of ecotype formation within species (Fine et al., 2013; Lowry et al., 2019). This ecotypic divergence may lead to distinct growth–defense strategies in congeners adapted to different habitats (e.g. Fine et al., 2004, 2006). However, to link ecotypic divergence in growth–defense strategies to species diversification, we must understand whether and how growth–defense trade‐offs can promote reproductive isolation. Sympatric, interfertile species provide an opportunity to study how growth–defense trade‐offs may contribute to reproductive isolation.

**FIGURE 1:**
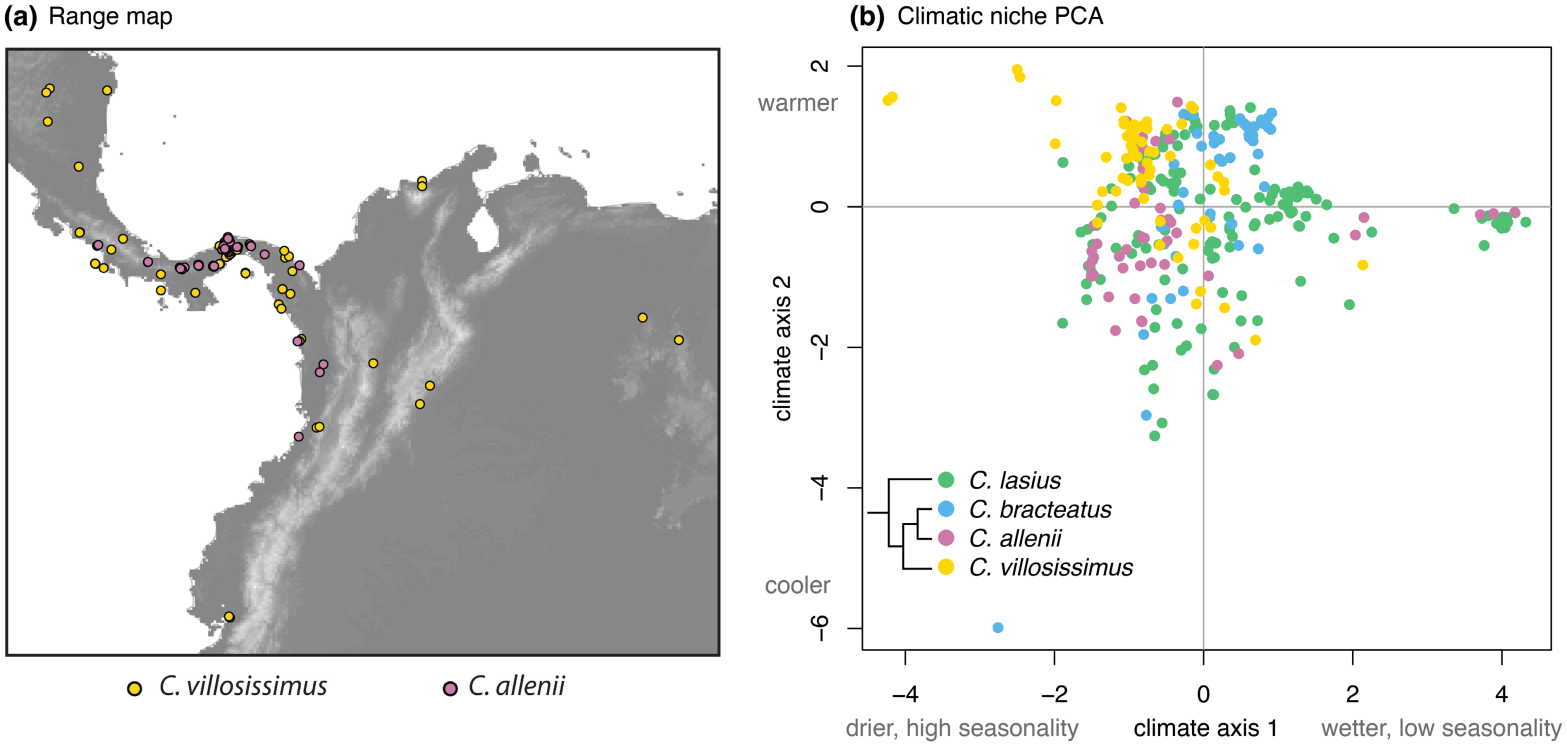
Range map and climate niches of the focal species and close relatives. (a) *Costus allenii*'s range falls entirely within that of *C. villosissimus* in Central America. Mapped occurrences of *C. allenii* are shown in pink, and *C. villosissimus* in yellow. (b) *Costus villosissimus* is generally found in a warmer, drier, and more seasonal climate niche than close relatives, including *C. allenii*, as shown by principal component analysis of climate from occurrence data. Principal component axes summarize four climate variables: mean annual temperature, mean annual precipitation, temperature seasonality, and precipitation seasonality. Climate axis 1 (PC1) explains 45.5% of the data variance while climate axis 2 (PC2) explains 27.1% of the variance. All climate data were projected and resampled to a 1 km^2^ grid cell size, and the realized niche position of each species was estimated by circumscribing each species' niche relative to all occupied niche space across Neotropical *Costus*. Occurrence data and modified mapping and PCA code originate from Vargas et al. (2020); see their publication data repository for further details on climate data standardization and occurrence data.

Here, we investigate growth–defense trade‐offs in co‐occurring, interfertile, recently diverged species to assess empirically whether divergent growth–defense trade‐offs facilitate reproductive isolation. Our focal species of Neotropical spiral ginger, *Costus villosissimus* (Maas) and *C. allenii* (Jacq.), co‐occur in parts of Central America and diverged recently enough that they can still hybridize in nature (Figure 1a; Chen, 2011; Vargas et al., 2020). In fact, most American *Costus* species can form viable hybrid offspring (Kay & Schemske, 2008), and this lack of intrinsic isolation makes prezygotic barriers critical for young species' maintenance. The prezygotic barrier responsible for the majority isolation between *C. villosissimus* and *C. allenii* is divergent habitat adaptation, as shown in reciprocal transplant studies (Chen, 2011; Chen & Schemske, 2015). Although the species co‐occur in Central America, *C. allenii* grows in low light, perennially wet forest understory, whereas *C. villosissimus* grows along high light, seasonally dry forest edges (Chen & Schemske, 2015). *Costus villosissimus* survives seasonal drought by losing leaves to prevent water loss as the dry season progresses, then rapidly regrowing at the onset of the wet season (Harenčár et al., 2022). Conversely, *C. allenii* grows more slowly, given its darker environment. Based on the theoretical and empirical work on growth–defense tradeoffs in other plants described above, we predict that the differences in growth strategies between these species are accompanied by differences in defense allocation.

We first evaluate whether these focal species exhibit growth–defense trade‐offs, predicting lower defenses in the faster‐growing species. To test this prediction, we characterized differences in chemical defenses and leaf toughness. We then evaluated the consequences of these defenses for herbivory. We used controlled feeding trials with the species' primary herbivore to test for herbivore preference. We also evaluated herbivory in experimental and natural conditions in the wild. We placed potted pairs of both species in an intermediate habitat near a third, beetle‐occupied species and quantified herbivory. We also quantified natural herbivory on wild plants in their respective habitats. The combination of these methods allows us to separate the impact of intrinsic plant defenses versus local habitat on herbivory rates, providing insights into the eco‐evolutionary dynamics governing the impact of growth–defense trade‐offs on reproductive isolation.

## 2 MATERIALS AND METHODS

### 2.1 Study system

#### 2.1.1 Neotropical spiral gingers

Neotropical spiral gingers (*Costus* L.; Costaceae) are large, perennial understory monocots that rapidly diversified in Central and South American tropical forests within the last roughly 3 million years (Vargas et al., 2020). *Costus villosissimus* and *C. allenii* diverged between approximately 0.4 and 1.4 million years ago (Vargas et al., 2020), and *C. allenii*'s range is entirely within that of *C. villosissimus* (Figure 1a). They are part of a small clade of four species, three of which occupy wet, aseasonal niches (Vargas et al., 2020). In contrast, *C. villosissimus* occupies a warmer, drier, and more seasonal niche (Figure 1b). These large‐scale patterns translate to local habitat differentiation where the species co‐occur (Chen, 2011; Chen & Schemske, 2015). In central Panama, *C. allenii* grows in perennially wet, dark forest understory, whereas *C. villosissimus* grows in periodically dry, high‐light forest edges (Chen & Schemske, 2015). Divergent adaptation to these different habitats results in strong reproductive isolation between these species, as demonstrated by reciprocal transplant experiments (Chen, 2011; Chen & Schemske, 2015). When reciprocally transplanted, *C. villosissimus* has extremely low survival in *C. allenii* habitat, and *C. allenii* cannot survive in *C. villosissimus* habitat (Chen & Schemske, 2015). *Costus villosissimus* is drought‐adapted and takes advantage of its higher light environment with rapid growth, having 50% faster growth than *C. allenii* in a growth chamber common garden (Harenčár et al., 2022).

#### 2.1.2 Specialist herbivores

The young rolled leaves at the shoot tips of *Costus* and other species of the order Zingibrales are the preferred habitat and food source of numerous species of rolled‐leaf beetles (*Cephaloleia*; Chrysomelidae). These beetles' diet breadth ranges from specialists that feed on a single species of Zingiberales to generalists that feed on multiple plant species from the same or multiple families of Zingiberales (García‐Robledo, Kuprewicz, et al., 2013). Rolled‐leaf beetles associated with *Costus* are Costaceae family specialists (Garcia‐Robledo et al., 2017). Previous studies show that different rolled‐leaf beetle species vary in feeding and oviposition preferences among species of Costaceae (García‐Robledo & Horvitz, 2012). Different rolled‐leaf beetle species show differences in larval survival, adult longevity, fecundity, and fitness when feeding on different species in the spiral ginger family (Costaceae; García‐Robledo & Horvitz, 2011). However, rolled‐leaf beetle feeding has not yet been studied for *C. villosissimus* and *C. allenii*.

The damage caused by rolled‐leaf beetles has a characteristic pattern of roughly 1 mm wide tracks (Figure S1). Beetles eat extensively, and multiple beetles might feed on one plant. Furthermore, *Costus* leaves will often fall off after extensive rolled leaf beetle damage (García‐Robledo, personal observation). We rarely encountered signs of damage from other herbivores on *C. villosissimus* and *C. allenii*, whereas rolled‐leaf beetle damage was common and extensive in some areas. This suggests that the primary form of selection via herbivory is through the strong and relatively constant effects of specialist rolled‐leaf beetles.

To identify the species of rolled‐leaf beetles attacking *C. villosissimus* and *C. allenii*, we collected beetles from both species and several additional species of *Costus* in central Panama. Cryptic species complexes are common in *Cephaloleia* rolled‐leaf beetles (García‐Robledo, Kuprewicz, et al., 2013). For this reason, in addition to morphological identifications, we used the DNA barcode Cytochrome c oxidase subunit I (COI) to identify potential cryptic species (Hebert et al., 2004). We collected nine beetles in 95% (v/v) ET‐OH. We followed the protocols described by García‐Robledo, Erickson, et al. (2013) and García‐Robledo, Kuprewicz, et al. (2013), removing one leg for DNA extraction and species delimitation. We compared DNA sequences with the *Cephaloleia* DNA barcode library (published in GenBank, accession nos. KU357054–KU358485). All DNA sequences are in GenBank (accession numbers PP85181–PP851824). Beetle collections are deposited in the Biodiversity Research Collections of the Department of Ecology and Evolutionary Biology, University of Connecticut. We collected two species of *Cephaloleia*, but one was a rare, small, unidentified species. We focused our beetle experiments on the other species, *Cephaloleia dorsalis*, which was very abundant. Although *Cephaloleia* includes cryptic species, at our study site *C. dorsalis* represents a single, easily identifiable species that feeds on both *C. villosissimus* and *C. allenii*.

#### 2.1.3 Study sites

All fieldwork was conducted in a region of sympatry in the Colón Province of Central Panama. Wild plants of each species from which we collected herbivory, toughness, and chemistry data were located at two sites: along Pipeline Road in Soberanía National Park (about 10 km northeast of Gamboa) and on the Barro Colorado Island Nature Monument. Both species occur at both sites, segregated by microhabitat as described above. We assessed rolled‐leaf beetle damage on both species in 2019, a dry year, and again in 2022, a wet year. In the dry season (Dec–Apr) preceding sampling, Gamboa received 4 mm of precipitation in 2019 and 137 mm in 2022 (the sum for both years does not include 12/05–12/13 due to missing data; Panama Canal Authority). Similarly, during the dry season preceding sampling on Barro Colorado Island, there was 173 mm of precipitation in 2019 and 634 mm over the same period in 2022 (Physical Monitoring Program of the Smithsonian Tropical Research Institute). We collected beetles for beetle feeding trials in both 2019 and 2022 from creeks transecting Pipeline Road.

### 2.2 Are leaf structural defenses lower for the fast‐growing species?

We measured leaf toughness as a proxy for structural defense. We measured leaf toughness on mature leaves of 18 *C. allenii* and 20 *C. villosissimus* in the field, and on rolled or barely unrolled young leaves of 8 *C. allenii* and 9 *C. villosissimus* grown in greenhouses at the University of California, Santa Cruz. We measured toughness with a Medio‐Line 40,300, 300 g max penetrometer (Pesola AG, Switzerland), which measures the force (g) required to puncture a 1 mm diameter hole in the leaf. For plants in the field, we measured the fourth leaf from the top of the stem, or the third leaf if the fourth was missing or too damaged for measurements. To ensure the pattern observed in mature leaves of wild plants was the same for young leaves, we measured toughness on leaves that were either still rolled or just barely unrolled, representing the age of leaf most commonly attacked by rolled leaf beetles. We placed each leaf between two sheets of plexiglass with a 5 mm hole through both and centered the penetrometer rod in the hole, maintaining the leaf at a constant tension. We took the average of toughness measures collected at three points transecting the middle of the leaf: one ca. 1 cm from the leaf edge, one ca. 1 cm from the midrib, and one centered between the other two points.

To determine whether leaf toughness differs between *C. allenii* and *C. villosissimus*, we first assessed normality for toughness values of each species separately with normal Q–Q plots and Shapiro–Wilk normality tests, finding that the data for mature leaves of both species best fit a lognormal distribution. We conducted an unpaired Welch's two‐sample *t*‐test on log‐transformed data. Since the log‐transformed data still did not perfectly fit a normal distribution, we additionally ran a nonparametric Wilcoxon rank‐sum test on untransformed data to validate the *t*‐test results. Both the parametric and nonparametric tests had the same significance level, so we only report the *t*‐test results here. For young leaf toughness, we had very low sample sizes and thus ran a nonparametric Wilcoxon rank‐sum test on untransformed data. We used R v4.2.0 for all analyses (R Core Team, 2022).

### 2.3 Does chemical investment differ between the slow‐ and fast‐growing species?

To assess potential differences in leaf chemical investment between *C. allenii* and *C. villosissimus*, we investigated three chemical compound classes with high concentrations across the *Costus* genus: steroidal saponins (aka steroidal glycosides), phenolics, and flavonoids (Graham & Farnsworth, 2010). Steroidal saponins and phenolics are mainly quantitative defense chemicals, meaning that their deterrence of herbivores increases with increasing chemical compound concentrations. Steroidal saponins are relatively uncommon and found chiefly in monocots. They exhibit various biological activities and play essential roles in plant defense (Hussain et al., 2019; Osbourn et al., 1998). Phenolics are common generalist defense chemicals that reduce plant palatability across various herbivores (Levin, 1971, p. 197; Rehman et al., 2012). Flavonoids are a subclass of phenolics that play a major role in protection from abiotic stressors, including UV radiation, temperature, and water stress (Agati et al., 2020; Di Ferdinando et al., 2012). We analyzed the total investment in these three compound classes for both species to understand potential differences in resource allocation to chemical defenses between our focal species. We also quantified the oxidative capacity of extractable metabolites, a proxy for the functional capacity of defensive metabolites. We collected one young and one mature leaf from seven individuals of each species along Pipeline Road in Soberania National Park in Gamboa, Panama, wiped them clean (including removal of any epiphytes), and dried the tissue in silica gel. Unfortunately, we did not have sufficient dry mass from these collections to analyze young and mature leaves independently, and we combined young and mature leaf tissue for chemical analysis. We believe this still represents the relative difference in defensive chemistry between species and encompasses what is experienced by beetles given that beetles do occasionally feed on mature leaves.

We assessed foliar steroidal saponin content after preliminary results using HPLC‐DAD and derivatized GC–MS analysis revealed the presence of steroidal saponins in both *Costus* species. Most notably, both taxa show relatively high concentrations of dioscin and its aglycone diosgenin. Therefore, we estimated the foliar saponin content in our samples using a modification of the vanillin–sulfuric acid method (Le et al., 2018). Here, 35 mg of each sample was extracted with 1.5 mL of 100% ethanol (dried with a molecular sieve) in a bead mill for 6 min at 6 m/s using three ceramic beads. We centrifuged samples for 5 min at 31,000 *g* and combined a 100 μL supernatant aliquot with 100 μL of 8% vanillin (in 100% dry ethanol; source: Tokio Chemical Company, JP.) and 200 μL of 72% sulfuric acid. After mixing the reaction, we incubated it in a drybath for 10 min at 60°C. We then quenched the reaction in ice for 10 min and transferred it to glass cuvettes to be measured in a spectrophotometer at 455 nm (Thermo Scientific Genesis 30). We calculated equivalents of dioscin (mg/g) using a 7‐point dioscin calibration curve (1.00, 0.50, 0.25, 0.125, 0.0625, 0.031, 0.0156 mg/mL; Source: Apexbio Technology LLC, USA).

To estimate the foliar phenolic content of our two focal species, we used a modified version of the Folin–Ciocalteu spectrophotometric method (Ainsworth & Gillespie, 2007). This approach quantifies the extractable phenolic compounds in terms of the gallic acid equivalents (GAE) in milligrams per gram of dry leaf material. We extracted 25 mg of freeze‐dried foliar tissue for each sample with 1.25 mL of 70% methanol for 15 min in a bead mill using three ceramic beads (speed: 6 m/s). We centrifuged extracts for 5 min at 21,000 rpm, then added aliquots of 20 μL of supernatant to 200 μL of 10% v/v Folin–Ciocalteu reagent (source: Sigma Aldrich Fine Chemicals, USA) and 1.25 mL of sodium carbonate (0.35 M). We vortexed samples for 1 min and incubated them in a dry bath for 15 min at 35°C. We then transferred samples to microcuvettes to measure absorbance at 765 nm. We conducted three analytical replicates for each sample and averaged the values to give the final value of each sample. We used gallic acid as a positive control and 70% methanol as a negative control. Data were transformed to mg/g using a 7‐point gallic acid calibration curve (0.25, 0.125, 0.062, 0.0312, 0.0156, 0.0078, 0.0039 mg/mL; source: Sigma Aldrich Fine Chemicals, USA). Because extractable flavonoids will also react positively to the Folin–Ciocalteu reagent, our foliar phenolic estimate will include foliar flavonoid content. Thus, to approximate relative differences between the two speices in the non‐flavonoid phenolic content, we subtracted our flavonoid content estimations from the final phenolic estimation.

To estimate flavonoid foliar content, we modified the aluminum complex spectrophotometric method. Initially proposed by Christ and Müller (1960), this approach relies on forming a complex between the aluminum ion and the carbonyl and hydroxyl groups of the flavonoid. Like the phenolic quantification, this approach estimates flavonoid content with standard equivalent units; here, we report flavonoid content as quercetin equivalent units in mg/g. We used 100 μL aliquots of the previous extraction (phenolics) for our samples. Each aliquot was combined with 1 mL of aluminum chloride (1% m/v), vortexed for 5 min, and incubated in a drybath for 15 min at 35°C. We then transferred samples to microcuvettes to measure absorbance at 415 nm. All samples had three analytical replicates that we averaged to yield sample values. We used quercetin as a positive control and 70% methanol as a negative control. Data were transformed to mg/g using a 7‐point quercetin calibration curve (0.25, 0.125, 0.062, 0.0312, 0.0156, 0.0078, and 0.0039 mg/mL).

We also used a 100 μL aliquot of the leaf tissue extractions to quantify the oxidative capacity of the metabolites extracted with a modified version of the 2,2‐diphenyl‐1‐picrylhydrazylradical (DPPH) radical scavenging activity (Blois, 1958). We combined aliquots with 500 μL of methanol and 500 μL of DPPH (0.195 mg/mL; Source: Sigma Aldrich Fine Chemicals, USA). We incubated samples in the dark at room temperature for 60 min. Samples were then transferred to microcuvettes and measured at 515 nm. All samples had three analytical replicates. We used ascorbic acid in methanol as a positive control and 100% methanol as a negative control. We transformed data to mg/g of ascorbic acid equivalents using a 7‐point curve (1.00, 0.50, 0.25, 0.125, 0.0625, 0.031, and 0.0156 mg/mL). While the relationship of oxidative capacity to insect herbivore deterrence has not been studied for *Costus* and rolled‐leaf beetles, the oxidative capacity of specialized metabolites has been shown to indicate insect herbivore deterrence in other systems (Appel, 1993; Bhonwong et al., 2009; Salminen & Karonen, 2011). The most cited mechanism for these negative effects on herbivores is the damage caused to gut membranes by the oxidation byproducts of phenolics at high pH, which is likely to impact rolled‐leaf beetles.

We compared oxidative capacity and chemical concentrations for each compound class between species. We assessed normality within species for oxidative capacity and each chemical compound class and found that the data were approximately normal. We conducted Welch's two‐sample *t*‐tests to compare the oxidative capacity and compound concentrations between species for each compound class (steroidal saponins, flavonoids, and non‐flavonoid phenolics). We used a false discovery rate *p*‐value adjustment for multiple tests (Benjamini & Hochberg, 1995).

### 2.4 Do beetles prefer the fast‐growing species in controlled feeding trials?

We conducted feeding trials in a controlled environment to understand beetle feeding preferences independent of the environment. In June 2019 and again in May and June 2022, we collected wild rolled‐leaf beetles (*Cephaloleia dorsalis*) from various species of *Costus* growing along creeks in the areas surrounding Pipeline Road. To ensure trial beetles were not biased towards one of our focal species, we did not collect beetles from either *C. villosissimus* or *C. allenii* and recorded the source plant species for inclusion as a random variable in our model. We transported beetles and conducted feeding trials following the methods of García‐Robledo and Horvitz (2012). We placed beetles in plastic ramekins with wet paper (20 lb white bond) lining the bottom and small holes in the lids. Beetles did not have access to leaves for 12 h before the feeding trials. We placed beetles in ramekins with one 1.5 cm^2^ leaf square each of *C. villosissimus* and *C. allenii*. After 12 h, we quantified the leaf area eaten of each species. We released beetles after each feeding trial, so each trial involved a fresh beetle. We conducted 13 successful feeding choice trials in 2019 and 27 in 2022. A successful trial was one in which the beetle ate anything; a trial was considered unsuccessful and removed if the beetle did not eat from either leaf square. To quantify herbivory, we laid a transparency printed with an mm^2^ grid over the leaf squares and counted mm^2^ of herbivore damage.

To evaluate the outcome of beetle feeding choice trials, we first standardized for individual beetle behavioral differences by subtracting the amount eaten of *C. allenii* from the amount eaten of *C. villosissimus* and dividing by the total amount eaten by the beetle. This calculation created a “preference” value ranging from −1 to 1, where negative values indicate a preference for *C. allenii*, positive values indicate a preference for *C. villosissimus*, and 0 indicates no preference. We then transformed these data to fit a beta distribution of values ranging from 0 to 1:

transformed preference = (preference − minimum)/(maximum − minimum) = (preference + 1)/2

The response variable data in a beta regression in R cannot include zeros or ones, so we added and subtracted 0.00001 to zeros and ones, respectively. This data modification is a conservative approach, as it makes the extreme values (0 or 1) less extreme. We performed a beta regression, with the transformed preference value as the response and trial date as a random effect. When we included beetle source plant species as a random variable, it did not describe any variability in the data or qualitatively change the results, so we removed it from the analysis.

### 2.5 Do beetles prefer the fast‐growing species in a natural environment with abundant beetles?

Beetle behavior in controlled feeding trials may not translate to natural environments where beetles experience complex ecological interactions and must expend energy and risk predation to fly between plants. To conduct feeding preference trials in the wild, we exposed greenhouse‐grown plants to a high beetle abundance natural habitat. We selected sites in a region where both focal species occur by finding beetle‐occupied individuals of a third *Costus* species, *C. scaber* along a stream. *Costus scaber* is common in streamside habitat and is frequently colonized by rolled‐leaf beetles. In 2022, we placed potted pairs of *C. allenii* and *C. villosissimus* about a meter from one another and a meter from a wild *C. scaber* exhibiting rolled‐leaf beetle damage. We paired plants to maximize similarity in size and number of stems, which corresponds to the number of young leaves on which beetles feed, with plants never differing by more than one stem. We recorded the presence or absence of rolled‐leaf beetle herbivory once a week and removed pairs that had experienced herbivory after 3 weeks to quantify herbivore damage. We replaced the removed pairs with new plant pairs and either moved or replaced pairs that did not experience any herbivory during their first 3 weeks in the field. We conducted three pair placement and evaluation rounds, with 11 pairs in the field per round. We moved (rather than replacing) two pairs between the first and second rounds and one (different) pair between the second and third rounds.

We used rolled‐leaf beetle herbivory presence/absence after 3 weeks to evaluate potential beetle preference for either species when presented with both in favorable habitat. First, we evaluated the impact of the specific locations where we placed potted pairs by fitting a binomial regression model with species as a fixed effect and local microsite as a random effect. Microsite, the locations where pairs of pots were placed, explained zero variability in the data, so we conducted a Pearson's Chi‐squared test with Yates' continuity correction.

We estimated the amount of rolled‐leaf beetle damage using the same method as feeding trials (overlaying an mm^2^ transparency grid and counting squares with herbivory). However, we could not obtain precise values for all plants due to post‐herbivory damage on some leaves (e.g., tearing and rotting). We analyzed data from the subset of plants from which we could get reasonable estimates. We fit a generalized linear mixed model with log‐transformed area of herbivore damage as the response, species as a fixed effect, and microsite as a random effect. We compared this to a model with no random effects with AIC values and a log‐likelihood ratio test, which both supported the simpler model. Therefore, we assessed whether herbivory varied between species with a paired Welch's two‐sample *t*‐test. The difference in herbivory between species in each pair was not normal, but nonparametric Wilcoxon rank‐sum test results did not differ from the *t*‐test, so we report only *t*‐test results here.

### 2.6 Does the fast‐growing species experience more or less herbivory in its natural habitat?

The differences in abiotic conditions between *C. allenii* and *C. villosissimus* habitat may impact the prevalence of rolled leaf beetles. To assess whether herbivory in the field was greater for faster‐growing *C. villosissimus*, we compared the presence and quantity of rolled‐leaf beetle herbivory between species in May and June for a dry (2019) and wet (2022) year. In 2019, we surveyed 52 *C. allenii* and 41 *C. villosissimus* for the characteristic patterns of rolled‐leaf beetle herbivory (Figure S1). In 2022, we surveyed another 97 *C. allenii* and 61 *C. villosissimus* for rolled‐leaf beetle herbivory.

In addition to recording the presence/absence of herbivory, we calculated the percent leaf area damaged by *Costus* specialist herbivores for a random subset of 10 individuals per species per year. We calculated the average leaf herbivory percentage from each stem's top four leaves. To measure leaf and herbivory area, we used ImageJ on photos of the leaves against a white background with a scale bar (ImageJ v1.53; Rasband, 1997; Schneider et al., 2012).

We used a binomial regression to assess whether species or year were significant predictors of rolled‐leaf beetle herbivory presence/absence. We evaluated the overall model fit and tested for under‐ or overdispersion of our model with the “DHARMa” package in R, finding none (Hartig & Lohse, 2022).

The average percent herbivory data were not normally distributed, so we evaluated whether species or year were significant predictors of herbivory with two nonparametric Wilcoxon rank‐sum tests, one testing for differences between years and the other between species.

## 3 RESULTS

### 3.1 Leaf structural and chemical defenses are lower in the faster‐growing species

The slower‐growing, wet, shade‐adapted species, *C. allenii*, invests more in toughness and specialized chemical compounds that contribute to defense in other systems (hereafter called “defense‐associated chemicals”; see below) than the faster‐growing, seasonally dry forest edge‐adapted species, *C. villosissimus*. Mature leaf toughness was about 31% greater on average for *C. allenii* than *C. villosissimus* (averages: *C. allenii* = 139 g, *C. villosissimus* = 106 g; *t*(33.24) = 3.624, *p* < .001), and this difference was even more pronounced in young leaves. Young *C. allenii* leaves were about 56% tougher on average than *C. villosissimus* leaves (averages: *C. allenii* = 127 g, *C. villosissimus* = 71 g; *W* = 71.5, *p* < .001). This aligns with previous findings of *C. allenii* having thicker (greater leaf mass per area) leaves (Harenčár et al., 2022).

**FIGURE 2:**
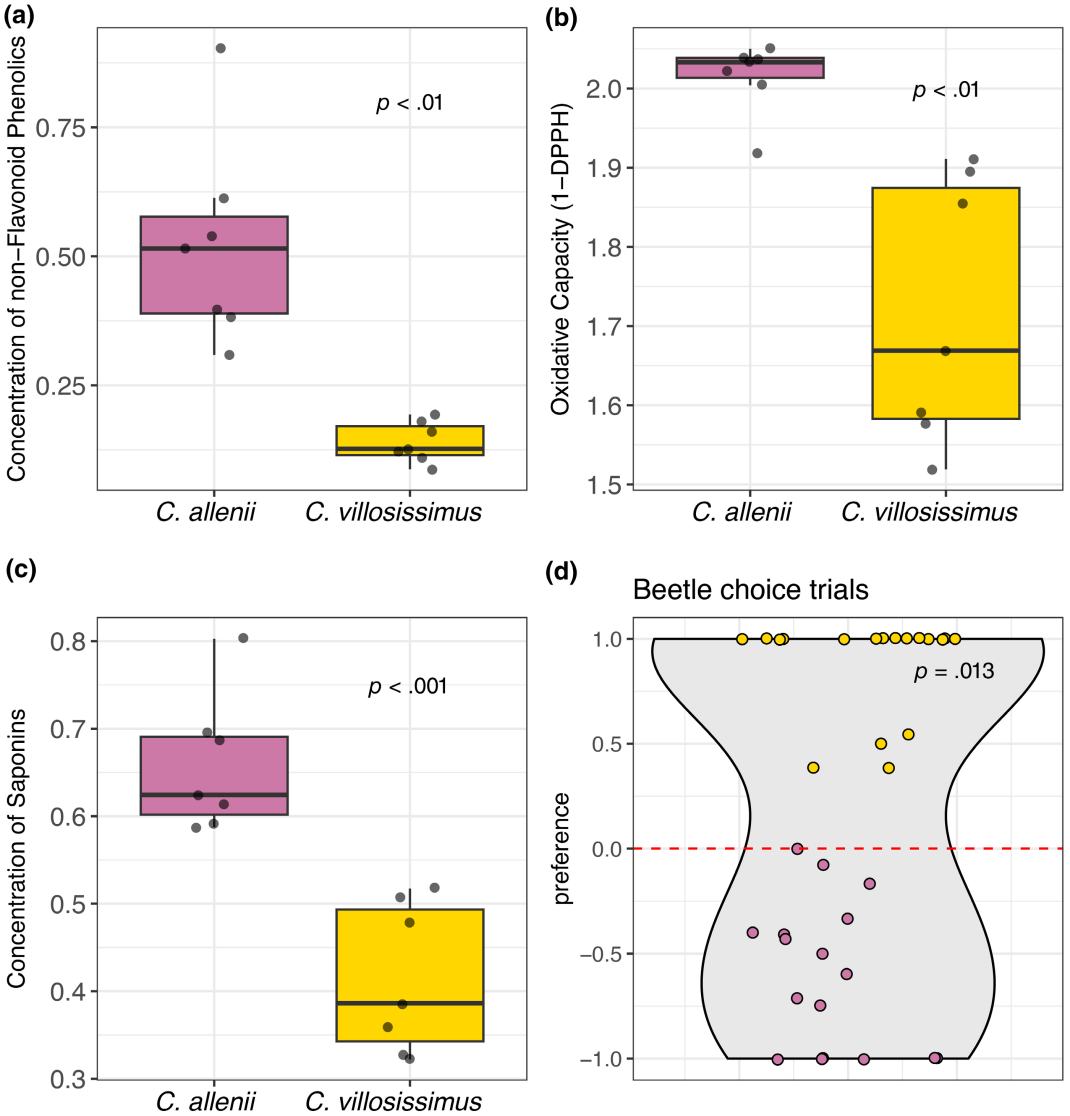
Leaf chemistry differences between *C. allenii* and *C. villosissimus*, and beetle preferences from controlled feeding trials. (a) Non‐flavonoid phenolics are common generalist defense chemicals. Units = GAEs in mg/g of dry leaf material. (b) Oxidative capacity represents the direct negative impact of non‐flavonoid phenolics on insects. Units = ascorbic acid equivalents in mg/g of dry leaf material. (c) Steroidal saponins include potent defense chemicals primarily found in monocots. Units = diocin equivalents in mg/g of dry leaf material. (d) Beetle preference from controlled feeding choice trials conducted in 2019 and 2022. Preference equals the difference between the amount eaten of 1.5 cm^2^ leaf squares of *C. allenii* and *C. villosissimus* divided by the total amount eaten by the beetle. Yellow dots above 0 indicate preference for *C. villosissimus*, and pink dots below 0 indicate preference for *C. allenii*. The gray‐shaded violin plot displays the kernel probability density, i.e. the width represents the proportion of data at that *y* value.

The overall chemical composition of *C. villosissimus* and *C. allenii* is similar. Still, they differ greatly in resource investment across the primary defense‐associated chemical compound classes (non‐flavonoid phenolics and steroidal saponins). On average, *C. allenii* has about 274% higher concentrations of non‐flavonoid phenolics (Figure 2a; *t*(6.471) = 5.025, adjusted *p* = .003), about 59% higher concentrations of steroidal saponins (Figure 2c; *t*(11.886) = 5.594, adjusted *p* = .0005), and about 17% higher oxidative capacity (Figure 2b; *t*(6.869) = 4.595, adjusted *p* = .003). The faster‐growing species, *C. villosissimus*, has greater investment in flavonoids. Although this diverse group of specialized metabolites can both deter and attract herbivores (Mierziak et al., 2014; Rosenthal & Berenbaum, 2012), they are better known for their potential role in the tolerance of abiotic stressors such as UV radiation and water stress (Agati et al., 2020; Di Ferdinando et al., 2012). *Costus villosissimus* has about 27% higher concentrations of flavonoids than *C. allenii* (Figure S2; *t*(7.708) = −3.966, adjusted *p* = .004). This result is in keeping with previous findings showing that *C. villosissimus* grows in forest edge habitat with higher light availability (mean light availability as % photosynthetically active radiation [PAR] of full sun for each habitat with 95% CI: *C. villosissimus* = 26.1% ± 4.9%, *C. allenii* = 11.3% ± 5.9%; Chen & Schemske, 2015), and periodic drought, necessitating drought adaptation (Harenčár et al., 2022). In summary, slow‐growing *C. allenii* invests more in defense‐associated chemicals, whereas fast‐growing, drought‐adapted *C. villosissimus* invests in chemical compounds that, in addition to potentially signaling or deterring herbivores, are likely to help the plant overcome abiotic stresses.

### 3.2 Beetles prefer the faster‐growing, less well‐defended species in controlled feeding trials

Beetles preferred *C. villosissimus* over *C. allenii* in controlled feeding choice trials. After controlling for date and differences in individual beetle behavior by calculating a standardized individual preference value for each beetle, we found that on average, individual beetles ate more *C. villosissimus* than *C. allenii* (average preference = 0.155, on a scale from −1 to +1, where a positive value indicated preference for *C. villosissimus*; Figure 2d; *β* ± SE = −1.268 ± 0.510, *p* = .013).

### 3.3 Both species are attacked at similar rates when placed in a natural environment with abundant beetles

**FIGURE 3:**
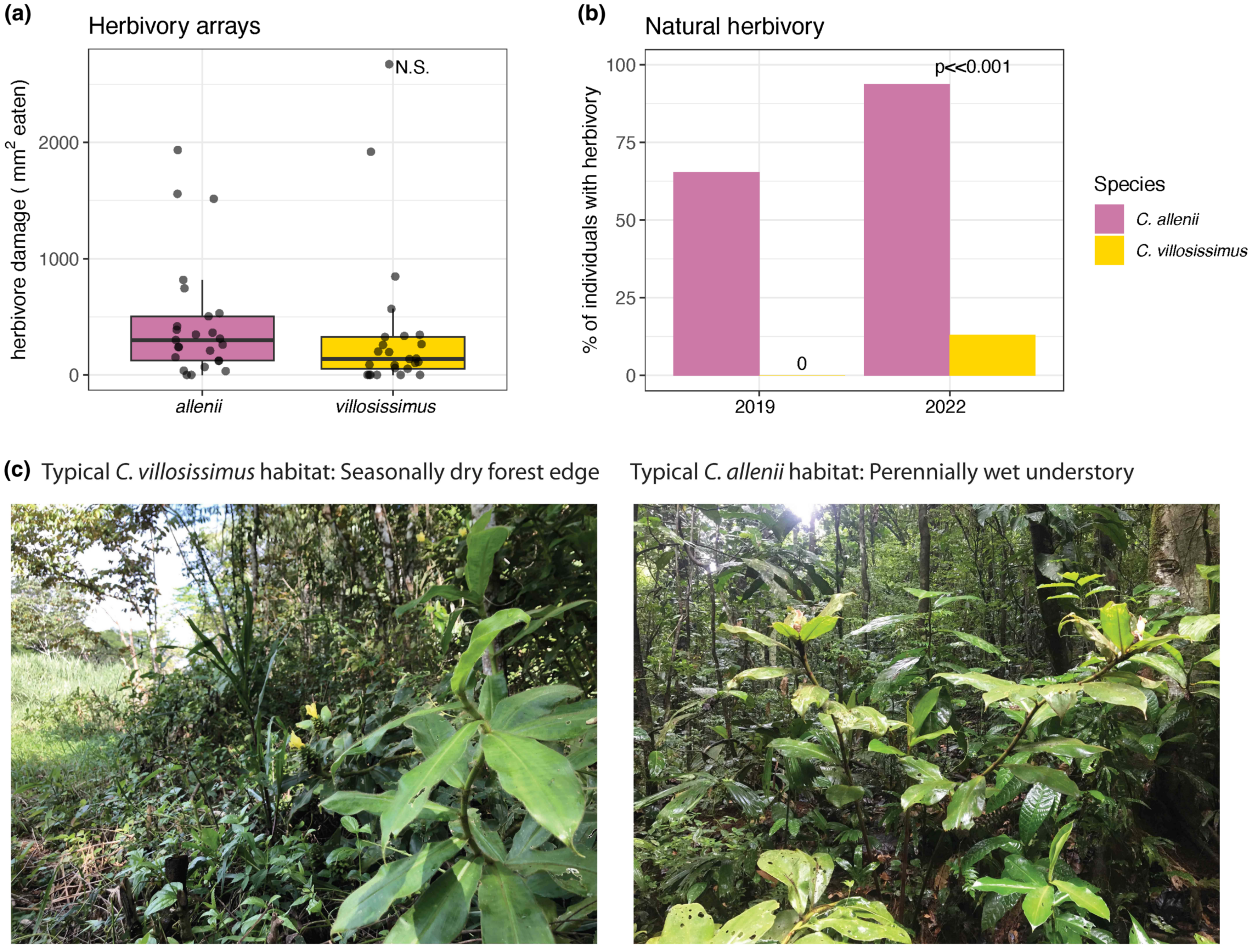
Patterns of rolled‐leaf beetle herbivory on *C. allenii* and *C. villosissimus* in (a) the experimental potted plants in beetle‐occupied habitat, and (b) in the wild, and (c) photographs of each species in representative habitat. (a) Estimated total amount of rolled‐leaf beetle damage on potted individuals of *C. allenii* and *C. villosissimus* set out beetle‐favorable, intermediate habitat: one individual of each species placed a meter from each other, and a meter from wild *C. scaber* occupied by at least one rolled‐leaf beetle. Box plots display median herbivory (black line) per species, interquartile range (box), and 1.5× interquartile range (whiskers). (b) Percentage of *C. allenii* and *C. villosissimus* individuals with rolled‐leaf beetle herbivore damage based on data from field surveys conducted in 2019 and 2022 in central Panama. (c) Photos of wild *C. villosissimus* (left) and *C. allenii* (right) in typical habitat; drier, higher light, and more open habitat for *C. villosissimus*, and wetter, lower light, and closed canopy habitat for *C. allenii*.

In contrast to the slight preference for *C. villosissimus* in controlled feeding trials, potted individuals of *C. allenii* and *C. villosissimus* placed in habitat with high beetle prevalence experienced similar frequency and amount of rolled‐leaf beetle damage. The Chi‐squared test on frequency (presence/absence) of herbivory did not show a significant difference, with a total of two *C. allenii* and six *C. villosissimus* out of 26 not experiencing herbivory (χ^2^ = 1.330, *p* = .249). The estimated area of herbivory also did not differ significantly between species (Figure 3c; *t*(23) = 0.778, *p* = .445).

### 3.4 There is less natural herbivory on the faster‐growing, less well‐defended species in its drier, higher‐light habitat

Again contrasting with controlled feeding trials and predictions based on differences in defense, the faster‐growing, less well‐defended *C. villosissimus* has less natural herbivory. Both presence/absence and percent herbivory data demonstrate that *C. allenii* experiences more rolled‐leaf beetle herbivory in the wild than *C. villosissimus*. In the presence/absence surveys, none of the *C. villosissimus* we visited had rolled‐leaf beetle damage in 2019, and few did in 2022, whereas most of the *C. allenii* did in both years (Figure 3a; *β* = −4.809, SE = 0.541, *z* = −8.890, *p* < .001). In the wetter year of 2022, while we still saw far more *C. allenii* with damage, rolled‐leaf beetle herbivory was more prevalent for both species (Figure 3a; *β* = 2.2435, SE = 0.493, *z* = 4.550, *p* <.001). Furthermore, the herbivory documented on *C. villosissimus* in 2022 was only found in the wetter microsites along Pipeline Road and never on plants along the roads further south where conditions are more open and drier.

Average percent herbivory across the subset of 10 individuals per species per year showed similar patterns. Average percent herbivory was 2.54% for *C. allenii* and 0% for *C. villosissimus* in 2019, and 3.06% for *C. allenii* and 0.19% for *C. villosissimus* in 2022. Percent herbivory of *C. allenii* was about 30 times greater than *C. villosissimus* (*W* = 372, *p* < .001). Plants experienced about 28% more herbivory in the wet year, but the difference between years was less apparent than in presence/absence data (*W* = 165, *p* = .3185), potentially due to the low sample size and high variability (Figure S3).

## 4 DISCUSSION

Growth–defense trade‐offs play a role in the habitat isolation of two closely related Neotropical spiral gingers. Rapid growth in *C. villosissimus* trades‐off with lower investment in leaf toughness and defense‐associated chemicals compared to *C. allenii*. *Costus allenii* invests in tougher leaves and higher concentrations of defense‐associated chemicals but grows more slowly (Figure 2). Differences in growth rate between the species align with differences in the abiotic habitat, where *C. villosissimus*' rapid wet season growth is part of its seasonal drought adaptation and is supported by higher light availability (Harenčár et al., 2022). The abiotic habitat differences also influence the biotic environment, with drier conditions reducing the prevalence of specialist beetle herbivores in *C. villosissimus* habitat.

Habitat differences rather than the differences in leaf defensive traits govern patterns of wild rolled‐leaf beetle herbivory between *C. allenii* and *C. villosissimus*. *Costus villosissimus* has lower investment in defense and is readily colonized and eaten by rolled‐leaf beetles in environments with prevalent beetles. In controlled choice trials, *C. villosissimus* experienced more herbivory than *C. allenii* (Figure 2d). However, both species experienced similar amounts of herbivory when placed together in wet, beetle‐occupied microsites. The lack of preference in these field‐based experiments could be due to low sample size relative to preference variability, but the results still suggest that environmental factors are important to beetle feeding behavior (Figure 3). The importance of environment becomes even more evident when looking at natural patterns of herbivory. *Costus villosissimus* rarely experiences rolled‐leaf beetle herbivory in its higher light, lower moisture habitat (Figure 3). Furthermore, we observed no rolled‐leaf beetle damage on *C. villosissimus* after a pronounced dry season and greater frequency of damage on both species after a wetter dry season (Figure 3). The few *C. villosissimus* that experienced herbivory in the wetter year were those growing in wetter microsites (sites closer to the wetter, northern end of Pipeline road; JGH, personal observation). These results suggest that beetle herbivore pressure is lower in drier, more open *C. villosissimus* habitat.

Previous research supports our findings that drier habitat reduces herbivore pressure; rolled‐leaf beetles, and other beetles in the Cassidinae, do poorly or cannot survive without high humidity and moisture (Auerbach & Strong, 1981; Linzmeier & Ribeiro‐Costa, 2013; McCoy, 1984; Santos & Benítez‐Malvido, 2012; Seifert, 1982; Strong, 1977a, 1977b). For example, where the dry season is long and severe in the Pacific North of Costa Rica, the rolled leaves of *Heliconia latispatha* are not occupied by any of the rolled‐leaf beetles (including species of *Cephaloleia*) that frequently eat the same species in wetter parts of Costa Rica (Strong, 1977a). In addition to dry environments harboring fewer rolled‐leaf beetles, low moisture can negatively impact feeding in *Cephaloleia*, likely reducing the amount of rolled‐leaf beetle herbivory in dry microsites (Auerbach & Strong, 1981). Another example of adaptation to drier habitat enabling escape from herbivores comes from a neotropical shrub, *Clidemia hirta*. *Clidemia hirta* survival in forest understory is increased 12% by spraying with insecticide, but the treatment has no effect on survival in open sites, indicating escape from insect herbivores in the open sites (DeWalt et al., 2004). These findings align with our own results showing lower herbivory on *C. villosissimus* in its relatively drier, more open habitat.

We did not see greater herbivory on the species with lower defense investment, *C. villosissimus*, even in high herbivore pressure habitat in our field‐based experiment (Figure 3). This contrasts with the classic predictions put forth by Coley et al. (1985) in their original description of growth–defense trade‐offs, and with the patterns of greater herbivory on the species with lower defense investment typically seen in tropical plants (Fine et al., 2006; Kursar & Coley, 2003). Lower wild herbivory on *C. villosissimus* in the field choice experiment also contrasts with the pattern of slightly greater beetle preference for *C. villosissimus* (although beetles often ate some of each species) observed in our controlled feeding choice trials. These contrasting results may be due to differences in the environment of each experimental setup: in controlled feeding choice trials, beetles have both species available in an area smaller than the size of a typical *Costus* leaf. In contrast, the potted plants in the field were spaced a meter apart. The lack of strong beetle preference in the field experiment aligns with optimal foraging theory (Emlen, 1966; MacArthur & Pianka, 1966), whereby it may be more energetically efficient to stay on any palatable plant they encounter than to continue flying between plants searching for the least well‐defended species. This possibility is further supported by mark‐recapture studies showing that rolled‐leaf beetles tend to fly to the closest available host plant rather than finding the most preferred plant (Johnson, 2004; Johnson & Horvitz, 2005). In nature, *Costus* individuals of different species are generally dispersed throughout the forest rather than clustered together. Flying long distances between plants may be energetically costly and increase the risk of predation by increasing the time spent outside the protection of rolled leaves. These potential costs to beetles of moving between plants could account for the lack of differential herbivory in our field‐based experiment, despite differences in the plant species' growth versus defense investment.

Growth–defense trade‐offs prevent plant species from investing heavily in both defense and growth. Because of this, they promote differentiation between species adapting to habitats that differ in selection on growth and defense. Both abiotic and biotic habitat differences appear to favor differentiation in growth–defense strategy between *C. allenii* and *C. villosissimus*. *Costus villosissimus*' adaptation to high‐light, periodically dry environments is unusual in the *Costus* genus (Vargas et al., 2020). Furthermore, *C. villosissimus*' three most closely related species are shade‐adapted, suggesting that it has undergone a habitat shift relative to the other species in its clade, adapting to higherlight and seasonal drought (Figure 1b). It has achieved this adaptation in part through greater investment in flavonoids, which are associated with protection from UV radiation and tolerance of water stress. Critically, *C. villosissimus* has also evolved flexible drought deciduousness, in which it limits water loss during drought through leaf drop, then takes advantage of high light during the wet season to rapidly regrow (Harenčár et al., 2022). Investment in this rapid growth is favored over investment in herbivore defense by both the abiotic light and drought conditions, and reduced biotic herbivore pressure. The reduced herbivore pressure enables *C. villosissimus* to trade investment in defense for rapid growth without incurring a high cost for being poorly defended. In this way, abiotic and biotic selection act synergistically to favor investment in growth over defense in *C. villosissimus* and strengthen habitat isolation from the darker, wetter, higher herbivory *C. allenii* habitat.

Habitat isolation is the primary reproductive isolating barrier between *C. allenii* and *C. villosissimus*, which are interfertile and share pollinators (Chen, 2011; Chen & Schemske, 2015). In reciprocal transplants, *C. allenii* mortality in *C. villosissimus* habitat mainly occurred during the dry season, suggesting that *C. allenii* is excluded from *C. villosissimus* habitat by a lack of drought adaptation (Chen & Schemske, 2015; Harenčár et al., 2022). On the other hand, *C. villosissimus* grows more slowly in *C. allenii*'s darker habitat than in its lighter home habitat, and its mortality was gradual (Chen & Schemske, 2015). When growing in darker habitats, plants with lower pest resistance may fail to produce new leaves fast enough to compensate for losses to herbivory and have lower survival (Coley et al., 1985). This mechanism likely contributes to the exclusion of *C. villosissimus* from *C. allenii* habitat, although transplants manipulating herbivore access are necessary to test this hypothesis definitively. In summary, *Costus allenii*'s slower growth is associated with its lack of drought adaptation, and *C. villosissimus*' lower defense investment may contribute to its poor survival in *C. allenii*'s higher beetle abundance habitat. Therefore, local niche adaptation mediated by growth–defense trade‐offs promotes the reproductive isolation of these recently diverged tropical plants, contributing to tropical plant diversity.

## 5 CONCLUSIONS

Growth–defense trade‐offs are often described as contributing to the remarkable diversity of tropical plants, but little work has empirically investigated how (Maron et al., 2019; Marquis et al., 2016; Moreira et al., 2015; Salazar & Marquis, 2012; Schemske et al., 2009). Our empirical findings link previous work on the role of growth–defense trade‐offs in ecotype formation with larger‐scale phylogenetic patterns of species diversity and plant defense traits. Work on ecotypes shows that growth–defense trade‐offs can drive divergent plant adaptation to habitats varying in herbivore pressure and resource availability (Fine et al., 2013; Lowry et al., 2019). Phylogenetic research finds defense divergence between co‐occurring species and congeners that are likely not interfertile (Becerra, 2007; Coley et al., 2018; Daly et al., 2022; Kursar et al., 2009; Salazar et al., 2016; Sedio et al., 2017; Vleminckx et al., 2018). Our study provides an important bridge between these previous studies by establishing the role of growth–defense trade‐offs in prezygotic reproductive isolation; we find that divergent growth–defense strategies governed by trade‐offs contribute to habitat isolation that is strong enough to maintain isolation between sympatric close relatives. We describe how eco‐evolutionary dynamics dictate the outcomes of growth–defense trade‐offs and contribute to tropical plant diversification.

## AUTHOR CONTRIBUTIONS

**Julia G. Harenčár:** Conceptualization (lead); data curation (lead); formal analysis (lead); funding acquisition (lead); investigation (lead); methodology (lead); project administration (lead); resources (lead); supervision (equal); writing – original draft (lead). **Diego Salazar‐Amoretti:** Investigation (supporting); methodology (supporting); resources (supporting); writing – review and editing (supporting). **Carlos García‐Robledo:** Funding acquisition (supporting); investigation (supporting); methodology (supporting); resources (supporting); writing – review and editing (supporting). **Kathleen M. Kay:** Conceptualization (supporting); funding acquisition (supporting); investigation (supporting); methodology (supporting); project administration (supporting); resources (supporting); supervision (equal); writing – review and editing (equal).

## FUNDING INFORMATION

This research was funded by the Jean H. Langenheim Endowed Graduate Fellowship, a Tinker Foundation Field Research Grant, the P.E.O International Scholar Award, the Garden Club of America Fellowship in Tropical Botany, the UCSC Ecology and Evolutionary Biology department's summer funding for graduate students, and National Science Foundation Dimensions of Biodiversity grant DEB 1737889 to K.M.K. and DEB 1737778 to C.G.‐R.

## CONFLICT OF INTEREST STATEMENT

We declare no conflicts of interest.

### OPEN RESEARCH BADGES

This article has earned Open Data, Open Materials and Preregistered Research Design badges. Data, materials and the preregistered design and analysis plan are available at https://github.com/jharencar/GDTO_article_analyses.

## Supporting information


Figure S1:

Figure S2:

Figure S3:

## Data Availability

All associated data and code are available on GitHub at https://github.com/jharencar/GDTO_article_analyses.

## References

[ece311609-bib-0001] Agati, G. , Brunetti, C. , Fini, A. , Gori, A. , Guidi, L. , Landi, M. , Sebastiani, F. , & Tattini, M. (2020). Are flavonoids effective antioxidants in plants? Twenty years of our investigation. Antioxidants, 9(11), 1098. 10.3390/antiox9111098 33182252 PMC7695271

[ece311609-bib-0002] Ainsworth, E. A. , & Gillespie, K. M. (2007). Estimation of total phenolic content and other oxidation substrates in plant tissues using Folin–Ciocalteu reagent. Nature Protocols, 2(4), 875–877. 10.1038/nprot.2007.102 17446889

[ece311609-bib-0003] Antonovics, J. (1976). The nature of limits to natural selection. Annals of the Missouri Botanical Garden, 63(2), 224–247. 10.2307/2395303

[ece311609-bib-0004] Appel, H. M. (1993). Phenolics in ecological interactions: The importance of oxidation. Journal of Chemical Ecology, 19(7), 1521–1552. 10.1007/BF00984895 24249181

[ece311609-bib-0005] Auerbach, M. J. , & Strong, D. R. (1981). Nutritional ecology of Heliconia herbivores: Experiments with plant fertilization and alternative hosts. Ecological Monographs, 51(1), 63–84. 10.2307/2937307

[ece311609-bib-0006] Becerra, J. X. (1997). Insects on plants: Macroevolutionary chemical trends in host use. Science, 276(5310), 253–256. 10.1126/science.276.5310.253 9092474

[ece311609-bib-0007] Becerra, J. X. (2007). The impact of herbivore–plant coevolution on plant community structure. Proceedings of the National Academy of Sciences of the United States of America, 104(18), 7483–7488. 10.1073/pnas.0608253104 17456606 PMC1855276

[ece311609-bib-0008] Benjamini, Y. , & Hochberg, Y. (1995). Controlling the false discovery rate: A practical and powerful approach to multiple testing. Journal of the Royal Statistical Society: Series B: Methodological, 57(1), 289–300.

[ece311609-bib-0009] Bhonwong, A. , Stout, M. J. , Attajarusit, J. , & Tantasawat, P. (2009). Defensive role of tomato polyphenol oxidases against cotton bollworm (*Helicoverpa armigera*) and beet armyworm (*Spodoptera exigua*). Journal of Chemical Ecology, 35(1), 28–38. 10.1007/s10886-008-9571-7 19050959

[ece311609-bib-0010] Blois, M. S. (1958). Antioxidant determinations by the use of a stable free radical. Nature, 181(4617), 1199–1200. 10.1038/1811199a0

[ece311609-bib-0011] Chen, G. F. (2011). *Experimental studies of adaptation and speciation in two neotropical* Costus *species* (Dissertation, Michigan State University).

[ece311609-bib-0012] Chen, G. F. , & Schemske, D. W. (2015). Ecological differentiation and local adaptation in two sister species of neotropical *Costus* (Costaceae). Ecology, 96(2), 440–449. 26240865 10.1890/14-0428.1

[ece311609-bib-0013] Christ, B. , & Müller, K. H. (1960). Zur serienmäßigen Bestimmung des Gehaltes an Flavonol‐Derivaten in Drogen. Archiv der Pharmazie, 293(12), 1033–1042. 10.1002/ardp.19602931202

[ece311609-bib-0014] Coley, P. D. , Bryant, J. P. , & Chapin, F. S. (1985). Resource availability and plant antiherbivore defense. Science, 230(4728), 895–899. 10.1126/science.230.4728.895 17739203

[ece311609-bib-0015] Coley, P. D. , Endara, M.‐J. , & Kursar, T. A. (2018). Consequences of interspecific variation in defenses and herbivore host choice for the ecology and evolution of Inga, a speciose rainforest tree. Oecologia, 187(2), 361–376. 10.1007/s00442-018-4080-z 29428967

[ece311609-bib-0016] Daly, D. C. , Perdiz, R. O. , Fine, P. V. A. , Damasco, G. , Martínez‐Habibe, M. C. , & Calvillo‐Canadell, L. (2022). A review of neotropical Burseraceae. Brazilian Journal of Botany, 45(1), 103–137. 10.1007/s40415-021-00765-1

[ece311609-bib-0017] DeWalt, S. J. , Denslow, J. S. , & Ickes, K. (2004). Natural‐enemy release facilitates habitat expansion of the invasive tropical shrub Clidemia Hirta. Ecology, 85(2), 471–483. 10.1890/02-0728

[ece311609-bib-0018] Di Ferdinando, M. , Brunetti, C. , Fini, A. , & Tattini, M. (2012). Flavonoids as antioxidants in plants under abiotic stresses. In P. Ahmad & M. N. V. Prasad (Eds.), Abiotic stress responses in plants: Metabolism, productivity and sustainability (pp. 159–179). Springer. 10.1007/978-1-4614-0634-1_9

[ece311609-bib-0019] Emlen, J. M. (1966). The role of time and energy in food preference. The American Naturalist, 100(916), 611–617. 10.1086/282455

[ece311609-bib-0020] Endara, M.‐J. , & Coley, P. D. (2011). The resource availability hypothesis revisited: A meta‐analysis. Functional Ecology, 25(2), 389–398. 10.1111/j.1365-2435.2010.01803.x

[ece311609-bib-0021] Endara, M.‐J. , Coley, P. D. , Ghabash, G. , Nicholls, J. A. , Dexter, K. G. , Donoso, D. A. , Stone, G. N. , Pennington, R. T. , & Kursar, T. A. (2017). Coevolutionary arms race versus host defense chase in a tropical herbivore–plant system. Proceedings of the National Academy of Sciences of the United States of America, 114(36), E7499–E7505. 10.1073/pnas.1707727114 28827317 PMC5594685

[ece311609-bib-0022] Fine, P. V. A. , Mesones, I. , & Coley, P. D. (2004). Herbivores promote habitat specialization by trees in Amazonian forests. Science, 305(5684), 663–665. 10.1126/science.1098982 15286371

[ece311609-bib-0023] Fine, P. V. A. , Metz, M. R. , Lokvam, J. , Mesones, I. , Zuñiga, J. M. A. , Lamarre, G. P. A. , Pilco, M. V. , & Baraloto, C. (2013). Insect herbivores, chemical innovation, and the evolution of habitat specialization in Amazonian trees. Ecology, 94(8), 1764–1775. 10.1890/12-1920.1 24015520

[ece311609-bib-0024] Fine, P. V. A. , Miller, Z. J. , Mesones, I. , Irazuzta, S. , Appel, H. M. , Stevens, M. H. H. , Sääksjärvi, I. , Schultz, J. C. , & Coley, P. D. (2006). The growth defense trade‐off and habitat specialization by plants in Amazonian forests. Ecology, 87(sp7), S150–S162. 10.1890/0012-9658(2006)87[150:TGTAHS]2.0.CO;2 16922310

[ece311609-bib-0025] García‐Robledo, C. , Erickson, D. L. , Staines, C. L. , Erwin, T. L. , & Kress, W. J. (2013). Tropical plant‐herbivore networks: Reconstructing species interactions using DNA barcodes. PLoS ONE, 8(1), e52967. 10.1371/journal.pone.0052967 23308128 PMC3540088

[ece311609-bib-0026] García‐Robledo, C. , & Horvitz, C. C. (2011). Experimental demography and the vital rates of generalist and specialist insect herbivores on native and novel host plants. Journal of Animal Ecology, 80(5), 976–989. 10.1111/j.1365-2656.2011.01843.x 21534952

[ece311609-bib-0027] García‐Robledo, C. , & Horvitz, C. C. (2012). Parent–offspring conflicts, “optimal bad motherhood” and the “mother knows best” principles in insect herbivores colonizing novel host plants. Ecology and Evolution, 2(7), 1446–1457. 10.1002/ece3.267 22957153 PMC3434947

[ece311609-bib-0028] Garcia‐Robledo, C. , Horvitz, C. C. , Kress, W. J. , Carvajal‐Acosta, A. N. , Erwin, T. L. , & Staines, C. L. (2017). Experimental assemblage of novel plant–herbivore interactions: Ecological host shifts after 40 million years of isolation. Biotropica, 49(6), 803–810. 10.1111/btp.12464 29398713 PMC5793930

[ece311609-bib-0029] García‐Robledo, C. , Kuprewicz, E. K. , Staines, C. L. , Kress, W. J. , & Erwin, T. L. (2013). Using a comprehensive DNA barcode library to detect novel egg and larval host plant associations in a Cephaloleia rolled‐leaf beetle (Coleoptera: Chrysomelidae). Biological Journal of the Linnean Society, 110(1), 189–198. 10.1111/bij.12115

[ece311609-bib-0030] Graham, J. G. , & Farnsworth, N. R. (2010). 3.04—The NAPRALERT database as an aid for discovery of novel bioactive compounds. In H.‐W. (Ben) Liu & L. Mander (Eds.), Comprehensive natural products II (pp. 81–94). Elsevier. 10.1016/B978-008045382-8.00060-5

[ece311609-bib-0031] Harenčár, J. G. , Ávila‐Lovera, E. , Goldsmith, G. R. , Chen, G. F. , & Kay, K. M. (2022). Flexible drought deciduousness in a neotropical understory herb. American Journal of Botany, 109, 1262–1272. 10.1002/ajb2.16037 35862815 PMC9545341

[ece311609-bib-0032] Hartig, F. , & Lohse, L. (2022). *DHARMa: Residual diagnostics for hierarchical (multi‐level / mixed) regression models* (0.4.5) [R]. https://CRAN.R‐project.org/package=DHARMa

[ece311609-bib-0033] Hebert, P. D. N. , Penton, E. H. , Burns, J. M. , Janzen, D. H. , & Hallwachs, W. (2004). Ten species in one: DNA barcoding reveals cryptic species in the neotropical skipper butterfly *Astraptes fulgerator* . Proceedings of the National Academy of Sciences of the United States of America, 101(41), 14812–14817. 10.1073/pnas.0406166101 15465915 PMC522015

[ece311609-bib-0034] Herms, D. A. , & Mattson, W. J. (1992). The dilemma of plants: To grow or defend. The Quarterly Review of Biology, 67(3), 283–335.

[ece311609-bib-0035] Hussain, M. , Debnath, B. , Qasim, M. , Bamisile, B. S. , Islam, W. , Hameed, M. S. , Wang, L. , & Qiu, D. (2019). Role of saponins in plant defense against specialist herbivores. Molecules, 24(11), 2067. 10.3390/molecules24112067 31151268 PMC6600540

[ece311609-bib-0036] Johnson, D. M. (2004). Source–sink dynamics in a temporally heterogeneous environment. Ecology, 85(7), 2037–2045. 10.1890/03-0508

[ece311609-bib-0037] Johnson, D. M. , & Horvitz, C. C. (2005). Estimating postnatal dispersal: Tracking the unseen dispersers. Ecology, 86(5), 1185–1190. 10.1890/04-0974

[ece311609-bib-0038] Kay, K. M. , & Schemske, D. W. (2008). Natural selection reinforces speciation in a radiation of neotropical rainforest plants. Evolution, 62(10), 2628–2642. 10.1111/j.1558-5646.2008.00463.x 18637960

[ece311609-bib-0039] Kursar, T. A. , & Coley, P. D. (2003). Convergence in defense syndromes of young leaves in tropical rainforests. Biochemical Systematics and Ecology, 31(8), 929–949. 10.1016/S0305-1978(03)00087-5

[ece311609-bib-0040] Kursar, T. A. , Dexter, K. G. , Lokvam, J. , & Coley, P. D. (2009). The evolution of antiherbivore defenses and their contribution to species coexistence in the tropical tree genus Inga. Proceedings of the National Academy of Sciences of the United States of America, 106(43), 18073–18078. 10.1073/pnas.0904786106 19805183 PMC2775284

[ece311609-bib-0041] Le, A. V. , Parks, S. E. , Nguyen, M. H. , & Roach, P. D. (2018). Improving the vanillin‐Sulphuric acid method for quantifying Total saponins. Technologies, 6(3), 84. 10.3390/technologies6030084

[ece311609-bib-0042] Levin, D. A. (1971). Plant phenolics: An ecological perspective. The American Naturalist, 105(942), 157–181.

[ece311609-bib-0043] Linzmeier, A. M. , & Ribeiro‐Costa, C. S. (2013). Seasonal pattern of Chrysomelidae (coleoptera) in the state of Paraná, southern Brazil. Biota Neotropica, 13(1), 153–162. 10.1590/S1676-06032013000100018

[ece311609-bib-0044] Lowry, D. B. , Popovic, D. , Brennan, D. J. , & Holeski, L. M. (2019). Mechanisms of a locally adaptive shift in allocation among growth, reproduction, and herbivore resistance in *Mimulus guttatus**. Evolution, 73(6), 1168–1181. 10.1111/evo.13699 30793293

[ece311609-bib-0045] MacArthur, R. H. , & Pianka, E. R. (1966). On optimal use of a patchy environment. The American Naturalist, 100(916), 603–609. 10.1086/282454

[ece311609-bib-0046] Maron, J. L. , Agrawal, A. A. , & Schemske, D. W. (2019). Plant–herbivore coevolution and plant speciation. Ecology, 100(7), e02704. 10.1002/ecy.2704 30916391

[ece311609-bib-0047] Marquis, R. J. , Salazar, D. , Baer, C. , Reinhardt, J. , Priest, G. , & Barnett, K. (2016). Ode to Ehrlich and raven or how herbivorous insects might drive plant speciation. Ecology, 97(11), 2939–2951. 10.1002/ecy.1534 27870033

[ece311609-bib-0048] McCoy, E. D. (1984). Colonization by herbivores of *Heliconia* spp. plants (Zingiberales: Heliconiaceae). Biotropica, 16(1), 10–13. 10.2307/2387887

[ece311609-bib-0049] Mierziak, J. , Kostyn, K. , & Kulma, A. (2014). Flavonoids as important molecules of plant interactions with the environment. Molecules, 19(10), 16240–16265. 10.3390/molecules191016240 25310150 PMC6270724

[ece311609-bib-0050] Moreira, X. , Abdala‐Roberts, L. , Parra‐Tabla, V. , & Mooney, K. A. (2015). Latitudinal variation in herbivory: Influences of climatic drivers, herbivore identity and natural enemies. Oikos, 124(11), 1444–1452. 10.1111/oik.02040

[ece311609-bib-0051] Osbourn, A. E. , Wubben, J. P. , Melton, R. E. , Carter, J. P. , & Daniels, M. J. (1998). Saponins and plant defense. In J. T. Romeo , K. R. Downum , & R. Verpoorte (Eds.), Phytochemical signals and plant‐microbe interactions (pp. 1–15). Springer US. 10.1007/978-1-4615-5329-8_1

[ece311609-bib-0052] R Core Team . (2022). R: A language and environment for statistical computing [computer software]. R Foundation for Statistical Computing. https://www.R‐project.org/

[ece311609-bib-0053] Rasband, W. S. (1997). ImageJ [computer software]. U.S. National Institutes of Health. https://cir.nii.ac.jp/crid/1573387450565680768

[ece311609-bib-0054] Rehman, F. , Khan, F. A. , & Badruddin, S. M. A. (2012). Role of phenolics in plant defense against insect herbivory. In L. D. Khemani , M. M. Srivastava , & S. Srivastava (Eds.), Chemistry of Phytopotentials: Health, energy and environmental perspectives (pp. 309–313). Springer. 10.1007/978-3-642-23394-4_65

[ece311609-bib-0055] Rosenthal, G. A. , & Berenbaum, M. R. (2012). Herbivores: Their interactions with secondary plant metabolites: The chemical participants. Academic Press.

[ece311609-bib-0056] Salazar, D. , Jaramillo, M. A. , & Marquis, R. J. (2016). Chemical similarity and local community assembly in the species rich tropical genus piper. Ecology, 97(11), 3176–3183. 10.1002/ecy.1536 27870051

[ece311609-bib-0057] Salazar, D. , & Marquis, R. J. (2012). Herbivore pressure increases toward the equator. Proceedings of the National Academy of Sciences of the United States of America, 109(31), 12616–12620. 10.1073/pnas.1202907109 22802664 PMC3411992

[ece311609-bib-0058] Salminen, J.‐P. , & Karonen, M. (2011). Chemical ecology of tannins and other phenolics: We need a change in approach. Functional Ecology, 25(2), 325–338. 10.1111/j.1365-2435.2010.01826.x

[ece311609-bib-0059] Santos, B. A. , & Benítez‐Malvido, J. (2012). Insect herbivory and leaf disease in natural and human disturbed habitats: Lessons from early‐successional Heliconia herbs. Biotropica, 44(1), 53–62. 10.1111/j.1744-7429.2011.00765.x

[ece311609-bib-0060] Schemske, D. W. , Mittelbach, G. G. , Cornell, H. V. , Sobel, J. M. , & Roy, K. (2009). Is there a latitudinal gradient in the importance of biotic interactions? Annual Review of Ecology, Evolution, and Systematics, 40(1), 245–269. 10.1146/annurev.ecolsys.39.110707.173430

[ece311609-bib-0061] Schneider, C. A. , Rasband, W. S. , & Eliceiri, K. W. (2012). NIH image to ImageJ: 25 years of image analysis. Nature Methods, 9(7), 671–675. 22930834 10.1038/nmeth.2089 PMC5554542

[ece311609-bib-0062] Sedio, B. E. , Rojas Echeverri, J. C. , Boya, P. C. A. , & Wright, S. J. (2017). Sources of variation in foliar secondary chemistry in a tropical forest tree community. Ecology, 98(3), 616–623. 10.1002/ecy.1689 27984635

[ece311609-bib-0063] Seifert, R. P. (1982). Neotropical Heliconia insect communities. The Quarterly Review of Biology, 57(1), 1–28.

[ece311609-bib-0064] Simms, E. L. , & Rausher, M. D. (1987). Costs and benefits of plant resistance to herbivory. The American Naturalist, 130(4), 570–581.

[ece311609-bib-0065] Sobel, J. M. , Chen, G. F. , Watt, L. R. , & Schemske, D. W. (2010). The biology of speciation. Evolution, 64(2), 295–315. 10.1111/j.1558-5646.2009.00877.x 19891628

[ece311609-bib-0066] Strong, D. R. (1977a). Insect species richness: Hispine Bettles of Heliconia Latispatha. Ecology, 58(3), 573–582. 10.2307/1939006

[ece311609-bib-0067] Strong, D. R. (1977b). Rolled‐leaf Hispine beetles (Chrysomelidae) and their Zingiberales host plants in middle America. Biotropica, 9(3), 156–169. 10.2307/2387878

[ece311609-bib-0068] Vargas, O. M. , Goldston, B. , Grossenbacher, D. L. , & Kay, K. M. (2020). Patterns of speciation are similar across mountainous and lowland regions for a neotropical plant radiation (Costaceae: *Costus*). Evolution, 74(12), 2644–2661. 10.1111/evo.14108 33047821

[ece311609-bib-0069] Vleminckx, J. , Salazar, D. , Fortunel, C. , Mesones, I. , Dávila, N. , Lokvam, J. , Beckley, K. , Baraloto, C. , & Fine, P. V. A. (2018). Divergent secondary metabolites and habitat filtering both contribute to tree species coexistence in the Peruvian Amazon. Frontiers in Plant Science, 9, 836. 10.3389/fpls.2018.00836 29971085 PMC6018647

[ece311609-bib-0070] Wink, M. (2003). Evolution of secondary metabolites from an ecological and molecular phylogenetic perspective. Phytochemistry, 64(1), 3–19. 10.1016/S0031-9422(03)00300-5 12946402

