# Supplementary figures and images for: Growth–defense trade‐offs promote habitat isolation between recently‐diverged species
Source: Ecol Evol. 2024 Jun 30;14(7):e11609. doi: 10.1002/ece3.11609 (PMC11214971; doi:10.1002/ece3.11609)

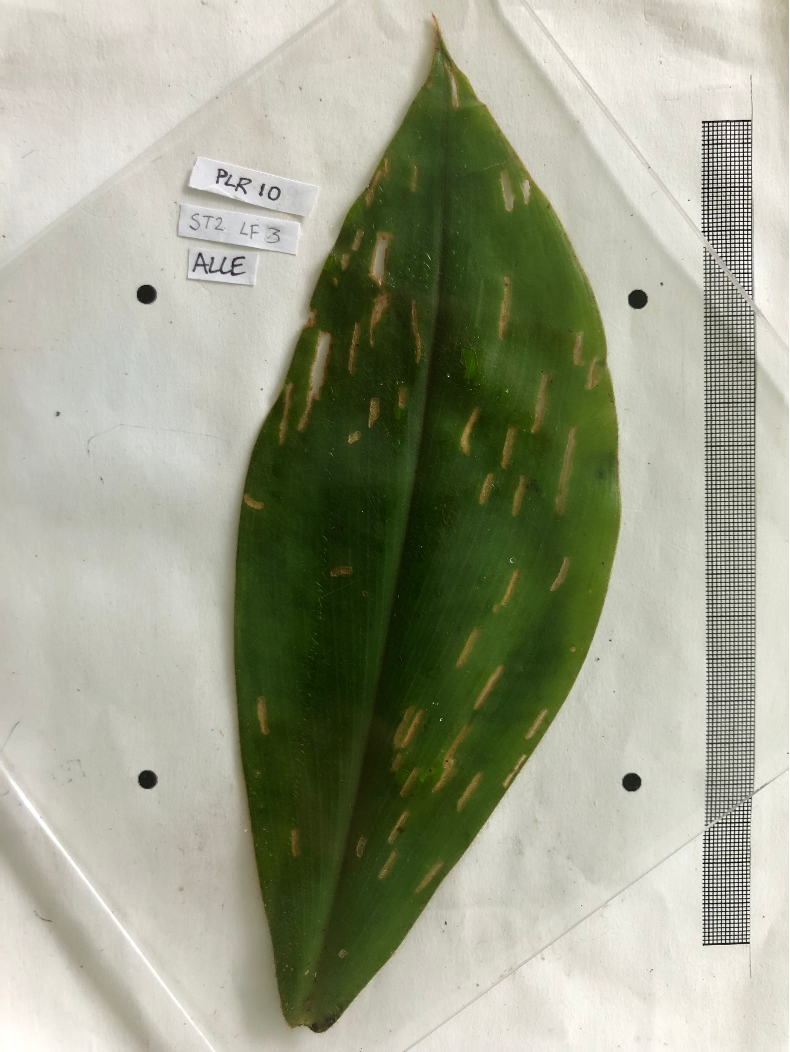

Supplement: Supplementary file 1 — Figure S1: [file ECE3-14-e11609-s002.png]

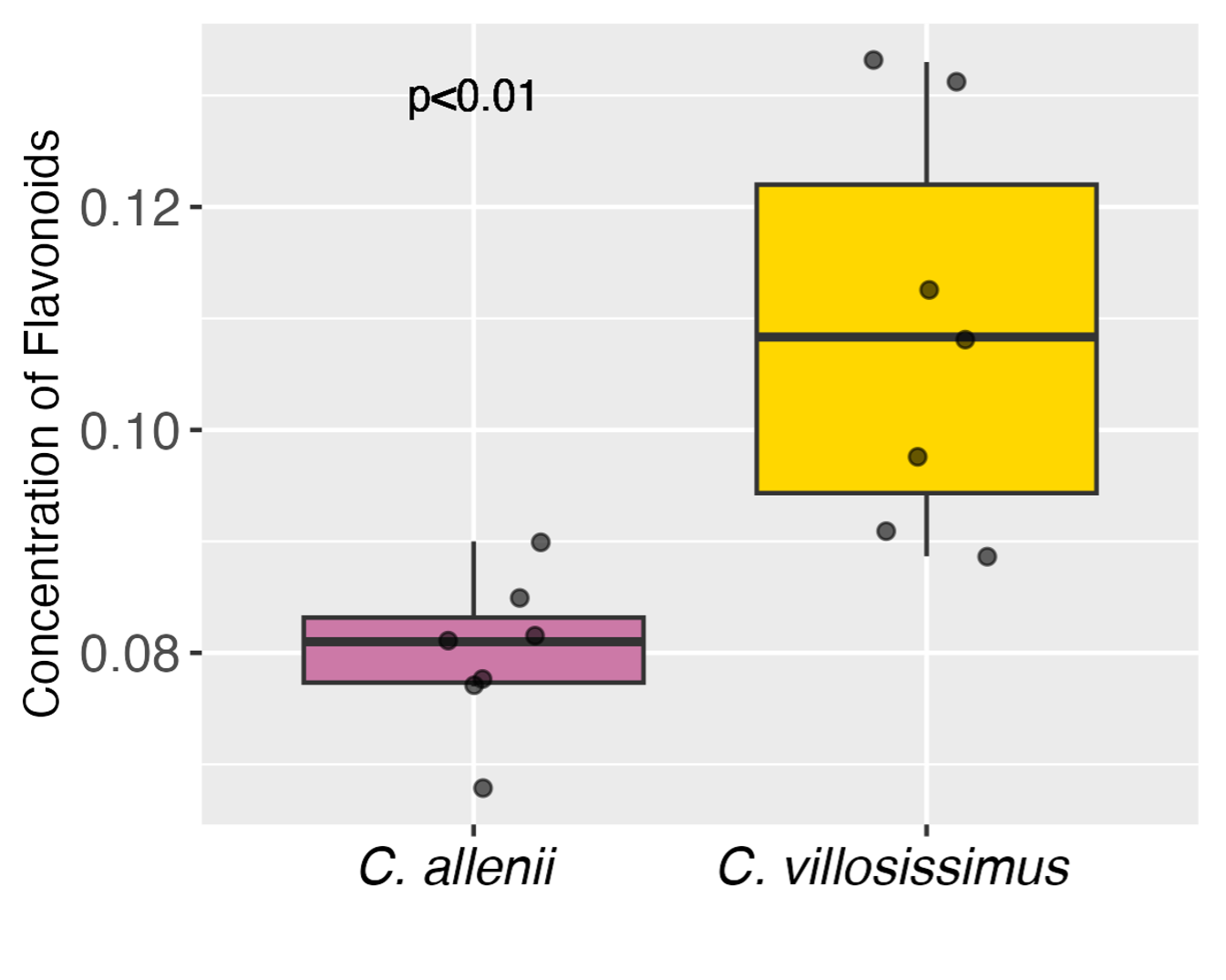

Supplement: Supplementary file 2 — Figure S2: [file ECE3-14-e11609-s001.png]

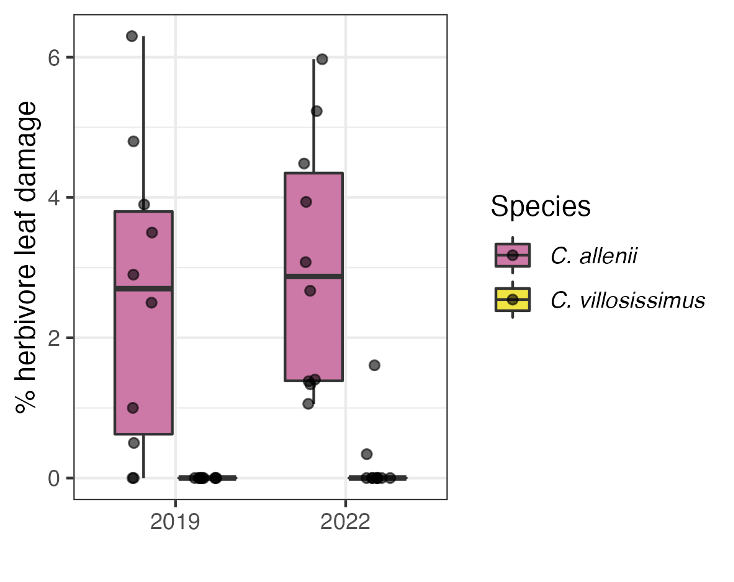

Supplement: Supplementary file 3 — Figure S3: [file ECE3-14-e11609-s003.png]
